# Elucidating the Hot Spot Residues of Quorum Sensing Peptidic Autoinducer PapR by Multiple Amino Acid Replacements

**DOI:** 10.3389/fmicb.2019.01246

**Published:** 2019-06-07

**Authors:** Avishag Yehuda, Leyla Slamti, Einav Malach, Didier Lereclus, Zvi Hayouka

**Affiliations:** ^1^Institute of Biochemistry, Food Science and Nutrition, The Hebrew University of Jerusalem, Rehovot, Israel; ^2^Micalis Institute, INRA, AgroParisTech, Université Paris-Saclay, Jouy-en-Josas, France

**Keywords:** quorum sensing, quorum quenching, PlcR antagonists, *B. cereus* group, anti-virulence peptides

## Abstract

The quorum sensing (QS) system of *Bacillus cereus*, an opportunistic human pathogen, utilizes the autoinducing PapR peptide signal that mediates the activation of the pleiotropic virulence regulator PlcR. A set of synthetic 7-mer PapR-derived peptides (PapR_7_; ADLPFEF) have been shown to inhibit efficiently the PlcR regulon activity and the production of virulence factors, reflected by a loss in hemolytic activity without affecting bacterial growth. Interestingly, these first potent synthetic inhibitors involved D-amino acid or alanine replacements of three amino acids; proline, glutamic acid, and phenylalanine of the heptapeptide PapR. To better understand the role of these three positions in PlcR activity, we report herein the second generation design, synthesis, and characterization of PapR_7_-derived combinations, alternate double and triple alanine and D-amino acids replacement at these positions. Our findings generate a new set of non-native PapR_7_-derived peptides that inhibit the PlcR regulon activity and the production of virulence factors. Using the amino acids substitution strategy, we revealed the role of proline and glutamic acid on PlcR regulon activation. Moreover, we demonstrated that the D-Glutamic acid substitution was crucial for the design of stronger PlcR antagonists. These peptides represent potent synthetic inhibitors of *B. cereus* QS and constitute new and readily accessible chemical tools for the study of the PlcR system. Our method might be applied to other quorum sensing systems to design new anti-virulence agents.

## Introduction

Quorum sensing (QS) is a cell-cell communication mechanism used to coordinate bacterial group behaviors (conjugation, virulence, sporulation, or competence) by assessing cell density through the production, secretion, and detection of small signaling molecules (Dunny and Leonard, 1997; Miller and Bassler, 2001; Slamti et al., 2014). Gram-negative bacteria appear to predominantly respond to *N*-acyl homoserine lactones, while QS in Gram-positive species mainly relies on the secretion of auto-inducing oligopeptides to bind and activate their cognate quorum sensors. In the past decade, a rapid increase of interest in bacterial quorum sensing peptides (QSPs) has emerged. Therefore, new QSPs databases are being established to provide chemical structures overview, microbial origin and functionality responses of these QS-derived signaling peptides (Gray et al., 2013; Wynendaele et al., 2013; Rajput et al., 2016).

The QSPs binding to their cognate quorum sensors occurs either on the outside of the bacterium (by interacting with a sensor in the membrane) or in the cytoplasm of the bacterial cell. In the latter case, the quorum-sensing regulators are controlled by direct interaction with a internalized signaling peptide (Dunny and Leonard, 1997; Lazazzera et al., 1997; Gominet et al., 2001; Miller and Bassler, 2001). They have been grouped in a new family of quorum sensors termed Rap-Rgg-NprR-PrgX-PlcR (RRNPP; Declerck et al., 2007; Neiditch et al., 2017). These quorum sensors are characterized by the presence of structural tetratricopeptide repeats (TPRs) forming a peptide binding domain (Blatch and Lässle, 1999), and a helix-turn-helix (HTH) DNA-binding domain (Wintjens and Rooman, 1996) in the case of transcriptional regulators. The PrgX – cCF10 system regulates conjugation in *Enterococcus faecalis* (Suzuki et al., 1984; Shi et al., 2005), the Rap phosphatases-Phr peptides system control competence and sporulation in *Bacillus subtilis* (Lazazzera et al., 1997; Perego and Brannigan, 2001; Perego, 2013), the transcriptional regulator/peptide pairs PlcR – PapR and NprR – NprX of the *Bacillus cereus* group are required for virulence and necrotrophism gene expression, respectively (Slamti and Lereclus, 2002; Perchat et al., 2011; Dubois et al., 2012; Grenha et al., 2013) and the archetype transcriptional regulator of the Rgg family, namely ComR that controls competence in most mutans, suis, pyogenes, bovis and salivarius *streptococci* (Mashburn-Warren et al., 2010; Fontaine et al., 2015) and predation in *S. salivarius* (Mignolet et al., 2018). The last discovered RRNPP transcriptional regulators are the PlcRa that activate the oxidative stress response and cysteine metabolism in transition state cells in *B. cereus* (Huillet et al., 2012) and aimR, which coordinates viruses of SPbeta group lysis-lysogeny decisions during infection of its *Bacillus* host cell (Erez et al., 2017).

The RRNPP family has an important role in adaptive and virulence processes in several bacteria (Slamti et al., 2014; Neiditch et al., 2017). This clearly identifies these regulators as major targets for the search of novel strategies against bacterial infections beyond conventional treatments. Antimicrobial therapy based on quorum quenching (QQ) can interfere or block all the processes involved in quorum sensing (Amara et al., 2011; Kalia, 2013; Grandclément et al., 2015). In contrast to antibiotics or antimicrobial agents, which aim at killing bacteria or inhibiting their growth, blocking cell-to-cell signaling mechanism, could attenuate bacterial pathogenicity without imposing the level of selective pressure on a bacterial population to develop resistance (Suga and Smith, 2003; Rasmussen and Givskov, 2006). A wide range of promising molecules have been already identified to inhibit QS-controlled virulence genes in Gram-negative bacteria (Hentzer et al., 2003; Galloway et al., 2012). On the other hand, except for strategies that have been investigated to inhibit the two component QS system Agr of *Staphylococcus*, which uses a peptide-thiolactone as the extracellular signal, the design of molecules modulating QS systems in Gram-positive bacteria has been poorly explored (Fontaine et al., 2010; Zheng et al., 2011; Tal-Gan et al., 2013a, 2014, 2016; Sully et al., 2014).

*Bacillus cereus* is a human opportunistic, Gram-positive spore-forming bacterial pathogen belonging to the *B. cereus* group (Stenfors Arnesen et al., 2008). This group comprises a number of highly phenotypically and genetically indistinguishable related species, including *Bacillus thuringiensis*, an insect pathogen, and *Bacillus anthracis*, the aetiological agent of anthrax (Helgason et al., 2000). The widespread presence of *B. thuringiensis* and *B. cereus* in soil and food, and their close relationship with *B. anthracis* make this group an important threat to public health (Rasko et al., 2005; Rossi et al., 2018), and a potential source of new pathogens. Indeed, *B. cereus* is generally regarded as a pathogen causing foodborne infections due to the production of enterotoxins such as Hbl and Nhe (Stenfors Arnesen et al., 2008), and nosocomial infections in an immuno-compromised patients (Granum and Lund, 1997; Kotiranta et al., 2000; Chu et al., 2001; Gaur et al., 2001; Bottone, 2010). *B. cereus* strains were also found to be responsible for severe infections resembling anthrax (Hoffmaster et al., 2004; Klee et al., 2006).

The QS system of *B. cereus* plays an important role in virulence (Agaisse et al., 1999; Gohar et al., 2008). *B. cereus* uses QS to establish infections by producing an arsenal of virulence factors, such as enterotoxins, pore-forming haemolysins, cytotoxins and various degradative enzymes (Granum and Lund, 1997; Vilas-Boas et al., 2002; Stenfors Arnesen et al., 2008; Ramarao and Sanchis, 2013). Production of most of these exported virulence factors is activated by PlcR, a 34 kDa protein that acts as a *B. cereus* group main virulence transcription factor (Lereclus et al., 1996; Agaisse et al., 1999; ØKstad et al., 1999; Gohar et al., 2008). Activity of PlcR depends on the binding of the signaling C-terminal heptapeptide PapR_7_ (ADLPFEF) at the end of the exponential growth stage. PapR_7_ is imported by the oligopeptide permease system (OppABCDF; Gominet et al., 2001), binds the tetratricopeptide repeat (TPR)-type regulatory domain of PlcR (Grenha et al., 2013) and promotes recognition of the PlcR box to transcriptional activation of the target genes (Lereclus et al., 1996; Gominet et al., 2001; Slamti and Lereclus, 2002; Bouillaut et al., 2008). This triggers a positive feedback loop that up-regulates the expression of *plcR*, *papR* and various virulence genes (Agaisse et al., 1999; Ivanova et al., 2003; Gohar et al., 2008).

The structural and molecular basis for the activation of PlcR by PapR has been the focus of several studies, which have revealed interesting insights on the PlcR – PapR interactions. The PlcR – PapR relationship has been shown to be strain specific; comparison of the amino acid sequences of PlcR and PapR from 29 different strains demonstrated the existence of four classes (I to IV) of PlcR – PapR pairs, defining four distinct pherotypes in the *B. cereus* group. While PapR sequences from different strains of the *B. cereus* group showed divergences in their three N-terminal residues, the PFEF core was more conserved (Slamti and Lereclus, 2005). In 2007, the crystal structure of the complex formed between the protein PlcR (from group I) and the C-terminal PapR_5_ pentapeptide (LPFEF) was published (Declerck et al., 2007). According to RRNPP conserved features, each subunit of PlcR is formed of an N-terminal HTH DNA-binding domain, and a C-terminal regulatory domain composed of five degenerated TPRs forming a peptide binding domain. Binding of PapR triggers an allosteric mechanism that leads to a drastic conformational change of the HTH domains upon the two half sites of the DNA binding site, known as PlcR-box. The LPFEF pentapeptide, PapR_5_ was identified as the minimal peptide size required for PlcR activation (Slamti and Lereclus, 2002). However, the physiologically relevant heptapeptide PapR_7_ displays a slightly better affinity for PlcR (Bouillaut et al., 2008; Grenha et al., 2013). In 2008, Bouillaut and co-workers established a molecular model for the complex formed between PlcR and the heptapeptide PapR_7_ based on the crystal structure of PapR_5_-bound PlcR (Declerck et al., 2007; Bouillaut et al., 2008). Structural analysis and directed mutagenesis of PlcR residues suggested that: a) activation of PlcR by PapR_7_ is triggered by the hydrophobic interactions of the leucine, and two phenylalanines with helices 5 and 7 of the TPR-containing domain of PlcR b) the central proline residue may be required for the PapR peptides to fit into the binding groove on PlcR and c) the glutamic acid of the FEF PapR_7_ core motif may function to selectively allow PapR to bind PlcR by ionic interactions with Lys87 and 89. In a follow up study in 2013, Grenha and co-workers, have determined the crystal structure of the ternary complex DNA-PlcR-PapR_7_. It has been reported that both PapR_7_ phenylalanine residues are located in hydrophobic pockets and the only specific interactions are made between the glutamate of PapR_7_ and residues Lys89, Gln237, and Tyr275 of PlcR.

Binding of PapR to PlcR is essential to trigger QS-mediated functions in *B. cereus*. Thus, we recently studied the PlcR – PapR activation in *B. cereus* and *B. thuringiensis* at the molecular level. We designed, synthesized and characterized synthetic PapR 7-mer derived peptides to determine the contribution of each residue within PlcR – PapR_7_ interactions. Our findings reveal the first set of non-native peptides that can repress the PlcR regulon and thus relevant virulence factors. Moreover, we could demonstrate that the repression is mediated by QS and regulation of PlcR expression without affecting bacterial growth (Yehuda et al., 2018). Interestingly, these first potent synthetic inhibitors involved D-amino acid or alanine replacements of either proline (P) glutamic acid (E) or phenylalanine (F) of the heptapeptide PapR (ADLPFEF). To better understand the role of these three crucial positions in PlcR activation, we report herein the second generation design, synthesis, and characterization of PapR_7_-derived combinations, alternate double and triple alanine and D-amino acids replacement at these positions. We propose this systematic replacement approach to elucidate other quorum quenching agents in Gram-positive bacteria.

## Materials and Methods

### Bacterial Strains and Growth Conditions

Bacterial strains used in this study: *B. thuringiensis* 407 Cry${}^{-}$ *plcA′Z* (Bt A’Z) and the PapR null-mutant 407 Cry${}^{-}$Δ*papR plcA*’*Z* (Bt ΔpapR A’Z) strains, containing a transcriptional fusion between the promoter of *plcA* and the *lacZ* reporter gene (as described previously; Gominet et al., 2001; Slamti and Lereclus, 2002); *B. cereus* strain ATCC 14579 (Ivanova et al., 2003). Unless otherwise noted, cells were grown in modified LB medium (16 g/L tryptone, 8 g/L yeast extract, 5 g/L NaCl) at 37°C and stored at −80°C in LB containing 25% glycerol. Kanamycin (200 μg/mL) was used for the selection of *B. thuringiensis.*

### Solid Phase Peptide Synthesis Methodology (SPPS)

All the peptides were synthesized using standard Fmoc-based solid-phase peptide synthesis (SPPS), microwave irradiation, procedures on Rink Amide resin (substitution 0.5 mmol/g, 25 μmol) in SPE polypropylene Single-Fritted tubes. The Fmoc-protecting group was then removed by treating the resin with 20% (v/v) piperidine diluted in dimethylformamide (DMF) followed by heating to 80°C in the microwave (MARS, CEM, United States; 2-min ramp to 80°C, 2-min hold at 80°C) with stirring. To couple each amino-acid, Fmoc-protected amino acids (4 equiv. relative to the overall loading of the resin), were dissolved in DMF and mixed with 2-(1H-benzotriazol-1-yl)-1,1,3,3-tetramethyluronium hexafluorophosphate (HBTU; 4 equiv.) and diisopropylethylamine (DIEA; 4 equiv.). The solution was allowed to pre-activate for 5 min before being added to the resin, and heated to 70°C in a multimode microwave (2-min ramp to 70°C, 4-min hold at 70°C) with stirring. After each coupling/deprotection cycle the resin was drained and washed with DMF (3 × 5 mL). Once peptide synthesis was completed, the peptide was cleaved from the resin, by mixing the resin with 3 mL cleavage cocktail of 95% trifluoroacetic acid (TFA), 2.5% triisopropylsilane (TIPS), and 2.5% deionized water for 3 h with agitation. The peptide mixture was precipitated from the TFA solution by the addition of cold ether and collected by centrifugation (Eppendorf R5810 8000 rpm for 10 min). The ether was then removed, and the peptide was dried under a stream of nitrogen, and lyophilized, before high-performance liquid chromatography (HPLC) purification.

### Peptide Purification

Crude peptides were purified and characterized with Reverse-Phase (RP)-HPLC. The crude peptides were diluted to a final concentration of 10 mg/ml in a solution of 20% acetonitrile (ACN) in water (v/v) or dimethyl sulfoxide (DMSO). A semi-preparative Phenomenex Kinetex C18 (5 μm, 10 × 250 mm) was used for preparative RP-HPLC work. An analytical Phenomenex Gemini C18 column (5 μm, 4.6 mm × 250 mm, 110 Å) was used for analytical RP-HPLC work (Supplementary Figure S1). Standard RP-HPLC conditions were as follows: flow rates = 5 mL min^–1^ for semi-preparative separations and 1 mL min^–1^ for analytical separations; mobile phase *A* = 18 MΩ water + 0.1% TFA; mobile phase *B* = ACN. Purities were determined by integration of peaks with UV detection at 220 nm using a linear gradient (first prep 5% B → 65% B over 60 min and second prep 26% B → 36% B over 20 min). The purity of the tested peptides was determined using a linear gradient (5% B → 65% B over 60 min). MALDI-TOF spectrometry (Bruker Daltonik, Germany) was used to validate the synthesized peptides molecular weight (Supplementary Table S1). The purified peptides were lyophilized and stored at −20°C.

### Analysis of PlcR Regulon Expression Using β-Galactosidase Assay

#### PlcR Activation Studies

Bt ΔpapR A’Z cells were grown overnight in LB medium with selective antibiotic. The cells were diluted 10^–3^ in modified LB to a final volume of 1 liter and incubated at 37°C with shaking (200 rpm) until onset of the stationary phase of bacterial growth (OD_600_ 3 ± 0.5). Various concentrations of synthetic peptides were added to 2 ml aliquots of culture, which were incubated for 1 h before centrifugation (Eppendorf centrifuge R5810, 4000 rpm for 5 min) and quantification of β-galactosidase.

#### Competition Studies of PapR_7_-Derived Peptides

Bt A’Z cells were grown overnight in LB medium. The cells were diluted 10^–3^ in modified LB to a final volume of 1 L and incubated at 37°C with shaking (200 rpm) until the end of the lag or late-exponential of bacterial growth (OD_600_ 0.1 ± 0.03; 1.8 ± 0.1, respectively). Different concentrations of synthetic peptides were added to 2 ml aliquots of culture and incubated for various times (1–24 h) before centrifugation (Eppendorf centrifuge R5810, 4000 rpm for 5 min) and quantification of β-galactosidase activity.

### β-Galactosidase Assay

β-galactosidase activity was measured as described previously (Yang et al., 2017), with minor modifications. Briefly, 200 μL aliquots from 2 ml treated cultures were added in triplicate to a clear 96-well microtiter plate, and then OD_600_ was measured and β-galactosidase activity was assayed. The final results were reported as percentage of activation, which is the ratio between the Miller units obtained after addition of the PapR_7_ analogs. In Bt ΔpapR A’Z strain, the plcA promoter activity was very low and considered as a baseline. In Bt A’Z strain, the untreated bacteria were considered as 100% of activation and the results were normalized accordingly. Each assay was repeated at least three times.

### Hemolytic Assay Toward Human Red Blood Cells

Bt A’Z or *B. cereus* ATCC 14579 cells were grown overnight in LB medium. The cells were diluted 10^–3^ in modified LB to a final volume of 1 liter and incubated at 37°C with shaking (200 rpm) until the end of the lag phase of bacterial growth (OD_600_ 0.1 ± 0.03). Different concentrations of synthetic peptides were added to 2 ml aliquots of culture and incubated for 2.5 h before centrifugation (Eppendorf centrifuge R5810, 4000 rpm for 5 min), separation and filtration (0.2 μm filter) of the supernatants of the treated cultures. Analyses of hemolytic activity were conducted as previously described using human red blood cells (Tal-Gan et al., 2013b; Lobel et al., 2015). Bacterial supernatants were serially diluted in Tris-buffered saline (pH 7.2, 10 mM Tris–HCl, 155 mM NaCl) with 1% human red blood cells (hRBC) suspension and were incubated for 30 min at 37°C. Hemolytic activities were measured by monitoring the absorbance at 420 nm.

### Statistical Analysis

Unless otherwise noted, the results are presented as the mean ± SEM. One-way analysis ANOVA of variance, followed by Tukey *post hoc* analysis was used for statistical analysis. The results were considered to be statistically significant if *p* < 0.01.

## Results

We have previously reported the first five synthetic peptidic inhibitors of *B. cereus* PlcR-PapR QS system; three independent alanine amino acid replacements (PapR_7_ – P4A, E6A, and F7A) and two D-amino acid substitutions (PapR_7_ – dE_6_ and dF_7_) showed great reduction of PlcR regulon expression and virulence factor secretion (Yehuda et al., 2018).

**FIGURE 1:**
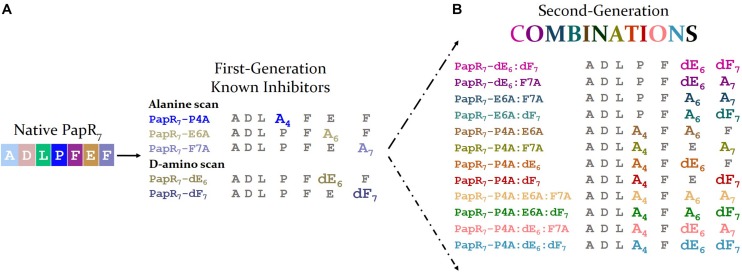
Second generation of PapR_7_-derived peptide combinations. **(A)** Sequences of first generation potent synthetic inhibitors 7-mer PapR-derived peptides (PapR_7_; ADLPFEF) involved D-amino acid or alanine replacements at three positions; proline (P) glutamic (E), and phenylalanine (F) of the heptapeptide PapR. **(B)** Sequences of second generation of PapR_7_-derived peptide combinations, involved double and triple alanine and D-amino acids replacement.

We initiated the current study by evaluating the three crucial positions of the heptapeptide PapR -proline (Pro4), glutamic acid (Glu6) and phenylalanine (Phe7) through systematic single or/and multiple amino acid substitution strategy. We designed, synthesized and purified a second generation set of PapR_7_-derived peptide combinations to further explore the structure–activity relationship delineated previously for the first-generation of peptidic analogs. This set included twelve peptides with double and triple alanine and D-amino acid replacements, at the crucial Pro4, Glu6 and Phe7 residues (Supplementary Table S1, Supplementary Figure S1 and Figure 1).

We scanned each of PapR_7_-derived peptide combinations for its ability to modulate the expression of the PlcR regulon using *B. thuringiensis* 407 Cry${}^{-}$ (Bt 407${}^{-}$) as a model bacterium for the *B. cereus* group. This strain cured of its plasmid is acrystalliferous and shows high phylogenic similarity with the *B. cereus* reference strain ATCC 14579 (Lereclus et al., 1989; Priest et al., 1994; Slamti and Lereclus, 2005). Two *lacZ*-based reporter strains were used in the current study, *B. thuringiensis* 407${}^{-}$ *plcA*′*Z* (Bt A’Z) and PapR-null mutant *B. thuringiensis* 407${}^{-}$Δ*papR plcA*′*Z* (Bt ΔpapR A’Z). Both reporter strains contain a transcriptional fusion between the *plcA* promoter region and the *lacZ* gene. *plcA* is a member of the PlcR regulon and its expression directly reflects the activity of PlcR. The activity of each PapR_7_-derived peptide combination was evaluated and compared to the previously described five *B. cereus* inhibitory synthetics PapR_7_-derived peptides (PapR_7_-P4A; E6A; F7A; dE_6_ and dF_7_; Yehuda et al., 2018).

**FIGURE 2:**
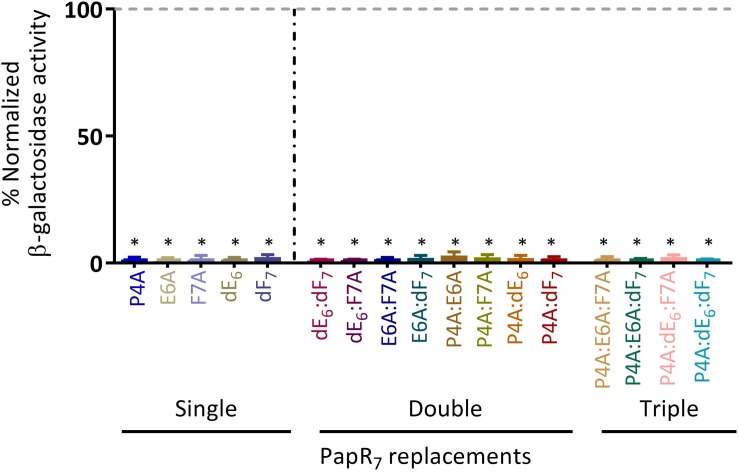
PlcR regulon activation by the new PapR_7_-derived peptide combinations. β-galactosidase activity of Bt ΔpapR A’Z induced by the addition of 5 μM PapR_7_-derived peptides normalized to synthetic PapR_7_ signal peptide at onset of the stationary phase of bacterial growth (OD_600_ of 3 ± 0.5, mean ± SEM, *n* = 9). ^*^p < 0.01 indicates a statistically significant difference between addition of synthetic PapR_7_ peptide and PapR_7_- derived peptides.

We first conducted an initial screening of all the analogs at high peptide concentration (5 μM) in order to evaluate their ability to activate the PlcR regulon to a level comparable to the synthetic PapR_7_ signal peptide (Figure 2). Both first and second generations of PapR_7_ analogous were classified by their number of amino acid replacements (alanine or D-amino); single, double and triple. Similar to our first-generation single replacement inhibitors, none of the new PapR_7_-derived peptide combinations were capable of activating the PlcR regulon. These findings revealed that any alanine and D-amino acid replacements, at the positions Pro4, Glu6 and Phe7 of PapR_7_ derivatives, are critical for PlcR regulon activation. These derivatives can therefore be classified as potential candidates for the development of potent second-generation PlcR inhibitors.

**FIGURE 3:**
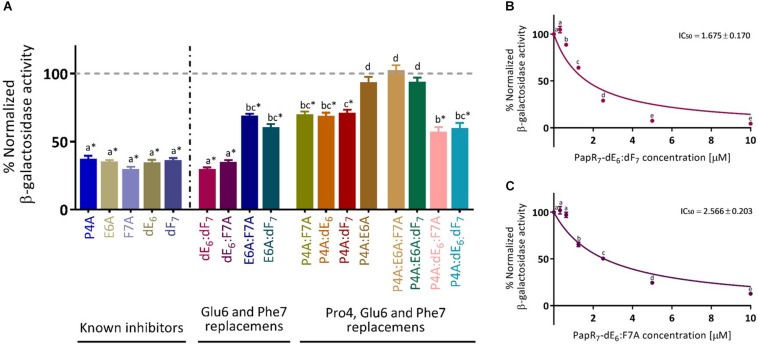
Competition studies with new PapR_7_-derived peptide combinations. **(A)** β-galactosidase activity of Bt A’Z induced by the addition of 2.5 μM PapR_7_-derived peptides, **(B)** PapR_7_- dE_6_:dF_7_, and **(C)** PapR_7_-dE_6_:F7A derivatives in several concentrations normalized to untreated bacterial cells at late-exponential of bacterial growth (OD_600_ of 1.8 ± 0.1, mean ± SEM, *n* = 9). ^*^p < 0.01 indicates a statistically significant difference between untreated Bt A’Z and addition of PapR_7_-derived peptides. Different letters indicate statistically significant differences between PapR_7_-derived peptide treatments (*p* < 0.01).

We next scrutinized their ability to compete with the endogenous PapR signal peptide (in Bt A’Z reporter strain) for reducing the activation of the PlcR regulon in late-exponential phase of bacterial growth (OD_600_ of 1.8 ± 0.1). As shown in Figure 3A; PapR_7_ derivatives activities were classified by D-amino acid enantiomer or Ala replacements at either of the three PapR_7_ crucial positions (Pro4, Glu6 and Phe7). The previously reported inhibitors, PapR_7_-P4A; E6A; F7A; dE_6_ and dF_7_, were also included for comparison. Peptides PapR_7_ – P4A:E6A, P4A:E6A:F7A and P4A:E6A:dF_7_ did not show any reduction in *plcA*’*Z* activity. PapR_7_ – E6A:F7A, E6A:dF_7_, P4A:F7A, P4A:dE_6_, P4A:dF_7,_ PapR_7_ – P4A:dE_6_:dF_7_ and P4A:dE_6_:F7A were able to reduce *plcA*’*Z* activity by approximately 40%. We identified two candidate peptides that inhibited *plcA*’*Z* activation when added at late-exponential phase. Indeed, PapR_7_ – dE_6_:dF_7_ and dE_6_:F7A reduced *plcA*’*Z* activity by 71 and 65 %, respectively, similarly to the inhibitory activity of their parent reporter inhibitors PapR_7_ – dE_6_, dF_7_ and F7A (65, 63, and 70%, respectively). To confirm PapR_7_ – dE_6_:dF_7_ and dE_6_:F7A potent inhibition, we repeated the experiment with several concentrations of these inhibitors in order to determine their IC_50_ values (Figures 3B,C). The results show that the new inhibitory peptides have IC_50_ values in the low micromolar range, almost comparable to their parent reporter inhibitors IC_50_ values (Table 1). Overall, we identified new potent inhibitors, PapR_7_ – dE_6_:dF_7_ and dE_6_:F7A, which were able to compete with endogenous PapR and inhibit PlcR regulon activity very effectively. Interestingly, these two potent inhibitors contain D-Glutamic acid replacement at position 6 of the PapR heptapeptide.

**TABLE 1:** Comparison between IC_50_ values of new PapR_7_-derived peptide combinations and their parent inhibitors, as determined by the *lacZ*-based reporter assay.

Ic_50_[μM]^*^			
PapR_7_-dE_6_:dF_7_	1.675 ± 0.170^a^	PapR_7_-dE_6_	0.977 ± 0.042^c^
PapR_7_-dE_6_:F7A	2.566 ± 0.203^b^	PapR_7_-dF_7_	1.223 ± 0.072^ac^
		PapR_7_-F7A	1.675 ± 0.102^a^

^*^IC_50_ values were calculated by GraphPad Prism 8, using the non-linear inhibitor vs. normalized response method. Different letters indicate statistically significant differences between IC_50_ values of PapR_7_-derived peptide combinations (*p* < 0.01).

**FIGURE 4:**
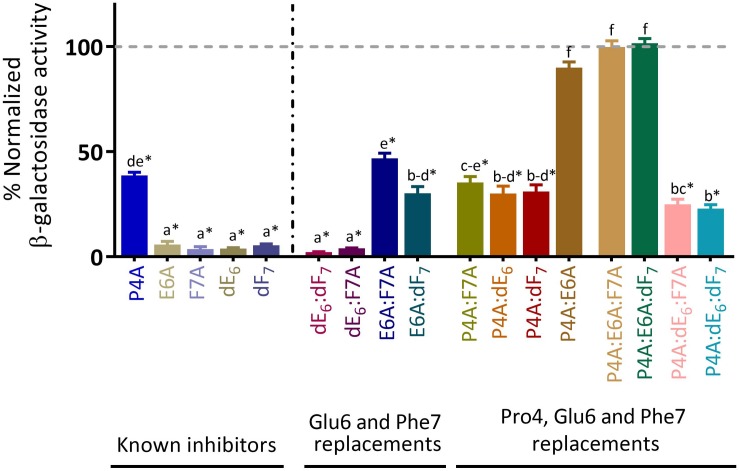
Exploring the effect of bacterial cell density on the PapR_7_-derived peptide combinations activity. β-galactosidase activity of Bt A’Z induced by the addition of 10 μM PapR_7_-derived peptides normalized to untreated bacterial cells at end lag phase of bacterial growth (OD_600_ of 0.1 ± 0.03; mean ± SEM, *n* = 9). ^*^p < 0.01 indicates a statistically significant difference between untreated Bt A’Z and addition of PapR_7_-derived peptides. Different letters indicate statistically significant differences between PapR_7_-derived peptide treatments (*p* < 0.01).

We have previously reported that inhibition through PapR_7_ inhibitory peptidic derivatives is cell density dependent (Slamti and Lereclus, 2002; Yehuda et al., 2018). To examine the effect of bacterial cell density on the inhibition of the PlcR regulon expression, each derivative was added to Bt A’Z cells at OD_600_ 0.1 ± 0.03, which corresponds to the early stage of exponential phase. PlcR-dependent gene expression was then quantified after 2.5 h (Figure 4) and after 24 h in order to assess their activity and stability over time (Supplementary Figure S2). As has been reported recently, all of our known parent PapR_7_ inhibitors were able to completely block *plcA*’*Z* activation for up to 24 h under these conditions (Yehuda et al., 2018). We observed similar results with PapR_7_ – dE_6_:dF_7_ and dE_6_:F7A; these peptides blocked *plcA*’*Z* activation compared to the parent peptidic inhibitors for 2.5 h and up to 24 h (Figure 4 and Supplementary Figure S2). Combining them with additional alanine replacement at Pro4, PapR_7_ – P4A:dE_6_:dF_7_ and P4A:dE_6_: F7A showed a reduction of ∼75% in *plcA*’*Z* activation. The addition of PapR_7_ derivatives as PapR_7_- E6A:F7A, E6A:dF_7_, P4A:F7A, P4A:dE_6_ and P4A:dF_7_ in the early stages of the bacterial growth led to drastic reduction in *plcA*’*Z* activity, revealing a series of new peptidic inhibitors. In contrast, the non-inhibitory peptidic combinations (PapR_7_- P4A:E6A, P4A:E6A:F7A and P4A:E6A:dF_7_) did not reduce the PlcR regulon expression even in low bacterial density. Importantly, the bacterial growth was not affected by the addition of all the examined peptides (data not shown).

Throughout these competition studies, we identified a group of three non-inhibitory peptidic combinations (PapR_7_- P4A:E6A, P4A:E6A:F7A and P4A:E6A:dF_7_), seven variants of PapR_7_ containing multiple replacements combinations with median to high inhibitory activities (PapR_7_- P4A:dE_6_:F7A, P4A:dE_6_:dF_7_, E6A:F7A, E6A:dF_7_, P4A:F7A, P4A:dE_6_ and P4A:dF_7_) and two very potent inhibitors that abolish activation of *plcA’lacZ* (PapR_7_ – dE_6_:dF_7_ and dE_6_:F7A) and compete with endogenous PapR. These two peptides are proposed as quorum quenchers that do not affect the bacterial growth but inhibit the expression of the PlcR regulon.

After observing this inhibitory activity, we expanded our study to explore the effect of these second generation PapR_7_ analogs on the production of a representative virulence factor under the control of PlcR in wild-type bacteria. Previous studies have shown that the activity of hemolysins in *B. cereus* is regulated by the PlcR – PapR QS system (Salamitou et al., 2000; Slamti and Lereclus, 2002; Slamti et al., 2004). Therefore, we studied the effect of the new PapR_7_ derivatives on the production of hemolysins in *B. cereus* strain ATCC 14579, a representative member of the *B. cereus sensu stricto* species.

**FIGURE 5:**
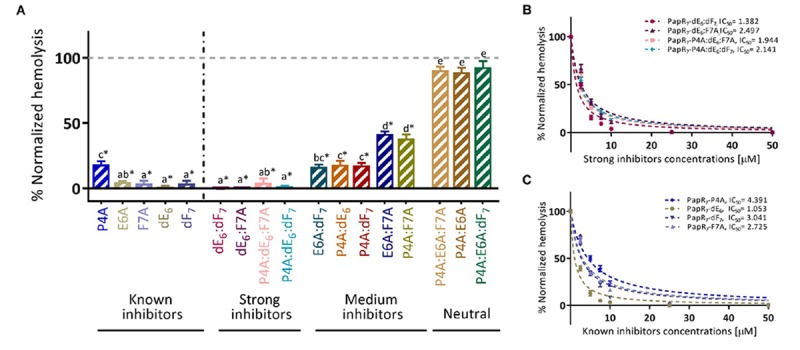
New PapR_7_-derived peptide combinations inhibit Bc virulence factor. **(A)** Hemolytic activity on human red-blood cells of supernatant *B. cereus* ATCC 14579 treated cultures in 10 μM of PapR_7_-derived peptides normalized to untreated bacterial cells at end lag phase of bacterial growth (OD_600_ of 0.1 ± 0.03; mean ± SEM, *n* = 9). Hemolysis inhibition dose response curves of *B. cereus* ATCC 14579 treated supernatant cultures in different concentrations of **(B)** PapR_7_ – dE_6_:dF_7_, dE_6_:F7A, P4A:dE_6_:dF_7_, and P4A:dE_6_:F7A and **(C)** known inhibitors normalized to untreated bacterial culture (mean ± SEM, *n* = 9). ^*^p < 0.01 indicates a statistically significant difference between untreated *B. cereus* ATCC 14579 and addition of PapR_7_-derived peptides. Different letters indicate statistically significant differences between PapR_7_-derived peptide treatments (*p* < 0.01).

**FIGURE 6:**
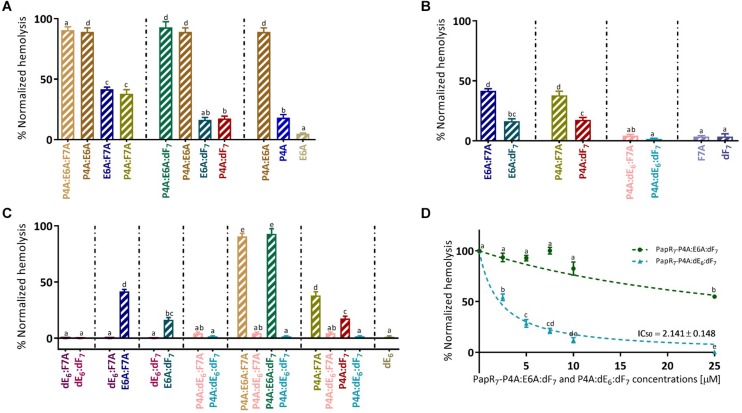
Exploring specific combination replacements effect on Bc virulence inhibition_._ Hemolytic activity of supernatant *B. cereus* ATCC 14579 treated cultures in 10 μM of PapR_7_-derived peptides normalized to untreated supernatants of bacterial cells at end lag phase of bacterial growth (OD_600_ of 0.1 ± 0.03; mean ± SEM, *n* = 9). Hemolytic activities on human red blood cells were classified by exploring the role of three PapR_7_ crucial positions in PlcR activity; **(A)** Role of individual and combined Pro4 or Glu6 residue replacements. **(B)** Effect of Phe7 replacement. **(C)** Importance of Glu6 and its stereoisomer substitution. Hemolysis inhibition dose response curves of *B. cereus* ATCC 14579 treated supernatant cultures in different concentrations of **(D)** PapR_7_ – P4A:E6A:dF_7_ and P4A:dE_6_:dF_7_ normalized to untreated bacterial culture (mean ± SEM, *n* = 9). Different letters indicate statistically significant differences between PapR_7_-derived peptide treatments (*p* < 0.01).

We performed hemolytic activity assays toward human red blood cells in the presence of the synthetic derivatives (Figure 5A). From these results, we identified PapR_7_ inhibitory peptidic analogs that were able to reduce the hemolytic activity of wild type *B. cereus* ATCC 14579. Interestingly, these analogs reduced the expression of hemolysin (Figure 5A) even more efficiently than the inhibition that was observed for Bt 407${}^{-}$ PlcR-dependent gene expression (as shown in Figure 4). We quantified the hemolytic activity of the strong inhibitors group by determining their IC_50_ values. In addition to the two strong inhibitors derivatives (PapR_7_ – dE_6_:dF_7_, dE_6_:F7A), we observed great activity also for PapR_7_ – P4A:dE_6_:dF_7_ and P4A:dE_6_:F7A analogs. Regard to PapR_7_ – dE_6_:dF_7_ and dE_6_:F7A derivatives, the relative IC_50_ value trends were highly similar to those in the *lacZ*-reporter assays results (Figure 5B and Tables 1, 2). In comparison to their parent single inhibitors PapR_7_ – dF_7_ and F7A, the relative IC_50_ values were in the same range (Figures 5B,C and Table 2); addition of D- Glutamic acid in the parent peptide PapR_7_ – dF_7_ (PapR_7_ – dE_6_:dF_7_) slightly improved its IC_50_ (IC_50_ value was reduced to 1.382 compared to 3.041), while alanine substituted at position Phe7 (PapR_7_ –dE_6_:F7A) did not show any effect. PapR_7_– P4A:dE6:dF7 and P4A:dE6:F7A exhibited lower IC_50_ values compared to their parent single inhibitors PapR_7_– P4A, dF_7_ and F7A but a higher IC_50_ value compared to the parent peptide PapR_7_–dE_6_ (Table 2). We observed another key feature in the new PapR_7_ combinations; sharing specific substitutions at PapR_7_ sequence influence their ability to inhibit PlcR activity; for example, all non-inhibitory peptidic combinations included alanine substitutions at positions Pro4 and Glu6 of PapR_7_ peptide, while all strong inhibitors group members share replacement of Glu6 by its D-isomer. All these new PapR_7_ combinations activity profiles may shed light on PlcR and PapR interaction. To better understand their role in PlcR activity, we divided the delineated above (Figure 5) PapR_7_-derived peptidic combinations hemolytic activities on human red blood cells to three different sets (Figures 6A–C).

**TABLE 2:** Comparison between IC_50_ values of new PapR_7_-derived peptide combinations and their parent inhibitors, as determined by the hemolytic assay.

IC._50_ values [μM]^*^			
PapR_7_-dE_6_:dF_7_	1.382 ± 0.117^ab^	PapR_7_-dE_6_	1.053 ± 0.089^a^
PapR_7_-dE_6_:F7A	2.497 ± 0.212^cd^	PapR_7_-dF_7_	3.041 ± 0.206^cd^
		PapR_7_-F7A	2.725 ± 0.239^d^
PapR_7_ - P4A: dE_6_: dF_7_	2.141 ± 0.148^b–d^	PapR_7_-P4A	4.391 ± 0.288^e^
PapR_7_-P4A: dE_6_: F7A	1.994 ± 0.184^a–c^	PapR_7_-dE_6_	1.053 ± 0.089^a^
		PapR_7_-dF_7_	3.041 ± 0.206^cd^
		PapR_7_-F7A	2.725 ± 0.239^d^

^*^IC_50_ values were calculated by GraphPad Prism 8, using the non-linear inhibitor vs. normalized response method. Different letters indicate statistically significant differences between IC_50_ values of PapR_7_-derived peptide combinations (*p* < 0.01).

## Summary

### Role of Individual and Combined Pro4 or Glu6 Residue Replacements

Replacing both Pro4 and Glu6 in PapR_7_ with alanine yielded non-active peptidic combinations regardless of other modifications in Phe7 position (PapR_7_- P4A:E6A, P4A:E6A:F7A, and P4A:E6A:dF_7_; Figure 6A). PapR_7_- **P4A:E6A**:F7A and **P4A:E6A** are non-inhibitory peptides, while PapR_7_- **E6A**:F7A and **P4A**:F7A defined as medium inhibitors, reduce the hemolytic activity by ∼60%. The same trend was observed with other two non-inhibitory peptidic combinations; PapR_7_- **P4A:E6A**:dF_7_ and PapR_7_- **P4A:E6A** compared to their disassembled peptidic combinations PapR_7_- **E6A**:dF_7_, **P4A**:dF_7_ and PapR_7_- **P4A**, **E6A,** respectively. These findings verified the dependence of proline and glutamic acid residues in PapR_7_ inhibitory activity. By modifying these two residues together to alanine the PapR_7_-derived inhibitors lost their antagonist features and their ability to prevent native PapR-PlcR interaction. This is in agreement with previous study (Bouillaut et al., 2008; Grenha et al., 2013), which emphasizes that both proline and glutamic acid have an important role in PlcR activation.

### Effect of Phe7 Replacement

Crystal structure of PapR_7_- PlcR complex showed that both PapR_7_ phenylalanine residues (Positions 5 and 7) are located in hydrophobic pockets (Grenha et al., 2013), and involved in hydrophobic interactions with PlcR. We examined the effect of replacing Phe7 with alanine or D-amino acid on the inhibitory activities of the designed peptides. Introducing D-Phenylalanine at position 7, regardless to the modifications at position 4 and 6, enhances the inhibitory activity of PlcR (Figure 6B). Replacing F7 in alanine (PapR_7_ – E6A:**F7A** or P4A:**F7A)** reduced hemolysis of red blood cells by 60%. However, these replacements combined with D-Phenylalanine yielded analogs (PapR_7_ – E6A:**dF_7_** and P4A:**dF_7_**) with stronger antagonistic activities (approximately 83% inhibition). These results indicate that the inclusion of D-Phenylalanine may contribute to hydrophobic interactions with PlcR by preserving the aromatic ring side chain interaction.

### Importance of Glu6 and Its Stereoisomer Substitution

We observed that all the strong inhibitor group members contain the replacement of Glu6 to its D-enantiomer (Figure 5A). PapR_7_ – **dE_6_**:F7A**** and **dE_6_**:dF_7_ displayed similar inhibitory effect, regardless of other modifications (D-amino or alanine replacements) in position Phe7 (Figure 6C). In contrast, replacing Glu6 with alanine yielded two weaker PlcR antagonists, when either Phe7 combined substitutions with alanine or D-amino (PapR_7_ – **E6A**:F7A and **E6A**:dF_7_). PapR_7_ – P4A:**dE_6_**:F7A and P4A:**dE_6_**:dF_7_ fully prevented hemolysis of red blood cells, however, replacing only Glu6 with alanine yielded two non-inhibitory peptidic combinations PapR_7_- P4A:**E6A**:F7A and P4A:**E6A**:dF_7_ as was supported by IC_50_ values (Figure 6D).

Interestingly, in an earlier study (Bouillaut et al., 2008; Grenha et al., 2013) the authors characterized the function and specific interactions of PapR glutamic acid with conserved residues in PlcR. These findings support our results about the important role of Glu6 in the activity of PlcR regulon. Indeed, replacement of L-glutamic acid of PapR_7_- P4A:F7A and P4A:dF_7_ (corresponding ADLAF**E**A and ADLAF**E**dF), with D-glutamic acid yielded two potent PlcR antagonists; PapR_7_- P4A:**dE_6_**:F7A and P4A:**dE_6_**:dF_7_. Overall, these three sets of new PapR_7_-derived peptide combinations support previous published studies and reveal the important role of three crucial positions at designing potent PlcR antagoinsts; Pro4, Phe7 and especially Glu6 that may function to selectively allow PapR, but not other similar autoinducers, to bind PlcR.

## Conclusion

The PapR-PlcR QS system is extensively involved in the pathogenesis of *B. cereus*, highlighting this system as an attractive target for an alternative treatment to prevent infection. We have previously reported the first five potent synthetic peptidic inhibitors of *B. cereus* PlcR-PapR QS system (Yehuda et al., 2018); three independent alanine amino acid replacements (PapR_7_ - P4A, E6A, and F7A) and two D-amino acid substitutions (PapR_7_ – dE_6_ and dF_7_). We concluded that the critical residues for PapR_7_ –PlcR interaction and PlcR activation were proline, glutamic acid and phenylalanine. To further understand their role in PlcR activity, a new set of PapR_7_ analogs with double and triple alanine and D-amino acid replacements at these positions were designed and synthesized. Multiple amino acid substitutions revealed that any replacement at these positions Pro4, Glu6 and Phe7 of PapR_7_ derivatives, is critical for PlcR regulon activation in the Δ*papR* mutant strain. A comprehensive competition study of all PapR_7_-derived peptides combinations in late-exponential phase identified four promising QS peptidic inhibitors candidates; PapR_7_ – dE_6_:dF_7_ and dE_6_:F7A and two other analogs with additional alanine substituted PapR_7_ – P4A:dE_6_:dF_7_ and P4A:dE_6_:F7A, all contained D-Glutamic acid at position 6 of the C-terminus of heptapeptide PapR. The two potent inhibitors PapR_7_ – dE_6_:dF_7_ and dE_6_:F7A generated similar inhibitory activity as their parent single replacement reported inhibitors PapR_7_ – dE_6_, dF_7_ and F7A with comparable IC_50_ values ≅ 1–2.6 μM. Our results verified previous reports that inhibition through PapR_7_ derivatives is cell density dependent (Slamti and Lereclus, 2002; Yehuda et al., 2018). We showed that all of our new four promising QS peptidic inhibitors candidates blocked the PlcR regulon activity even after 24-h period, when they were added at an early stage of bacterial growth (PapR_7_ – dE_6_:dF_7_, dE_6_:F7A, PapR_7_ – P4A:dE_6_:dF_7_, and P4A:dE_6_:F7A). Moreover, by exposing the bacterial cells to these analogs at earlier stage (OD_600_ 0.1 ± 0.03) we discovered a new series of inhibitors (PapR_7_- E6A:F7A, E6A:dF_7_, P4A:F7A, P4A:dE_6_, and P4A:dF_7_). Similar to the parent peptidic inhibitors, we hypothesized that the positive autoregulatory loop was blocked and quorum quenching was achieved throughout growth by the inhibitory multiple combinations PapR_7_ derivatives.

We next used a human red-blood cells hemolytic assay as a direct method to assess a QS-related phenotype linked to virulence in wild-type *B. cereus*. The inhibitory PapR_7_ peptidic analogs identified using the lacZ-reporter assays were even more efficient in reducing the hemolytic activity of wild type *B. cereus* ATCC 14579.

Our findings both corroborate and extend previous observations regarding the role of the PapR_7_ in PlcR receptor recognition; first, we showed the important role of proline or glutamic acid residues in PapR- PlcR interactions and as key in designing strong inhibitors. Second, we demonstrated that inclusion of D-Phenylalanine at Phe7 contribute to PapR_7_ derivatives inhibitory activities probably due to its hydrophobic features. Moreover, by interfering this Glu6 specific interactions with PlcR, we found the potential of D-Glutamic substitution at designing potent PlcR antagonist. These findings are consistent with previous study (Slamti and Lereclus, 2005) which investigated specificity and polymorphism of PlcR – PapR in the *B. cereus* group. Interestingly, while all the PapR sequences from different strains of the *B. cereus* group showed divergences in their three N-terminal residues, the E6 position was conserved. In the current study we highlighted the precise and unpredictable engineering of natural pheromone in our effort to develop new Quorum Quenching agents, reflecting the trade-off between good peptide binding and lower activation. These new non-native peptides inhibitors may be applied as chemical tools to further study the role of PlcR and other QS in all *B. cereus* group members. Further, our method of single and multiple amino acid replacements might be applied to other QS system to design new anti-virulence agents.

## Data Availability

No datasets were generated or analyzed for this study.

## Author Contributions

AY, LS, and EM performed the research. DL and ZH analyzed the data and wrote the manuscript.

## Conflict of Interest Statement

The authors declare that the research was conducted in the absence of any commercial or financial relationships that could be construed as a potential conflict of interest.
