# Supplementary material for: Elucidating the Hot Spot Residues of Quorum Sensing Peptidic Autoinducer PapR by Multiple Amino Acid Replacements
Source: Front Microbiol. 2019 Jun 7;10:1246. doi: 10.3389/fmicb.2019.01246 (PMC6568020; doi:10.3389/fmicb.2019.01246)
Supplement: Supplementary file 1 [file Data_Sheet_1.docx]

Supplementary Material


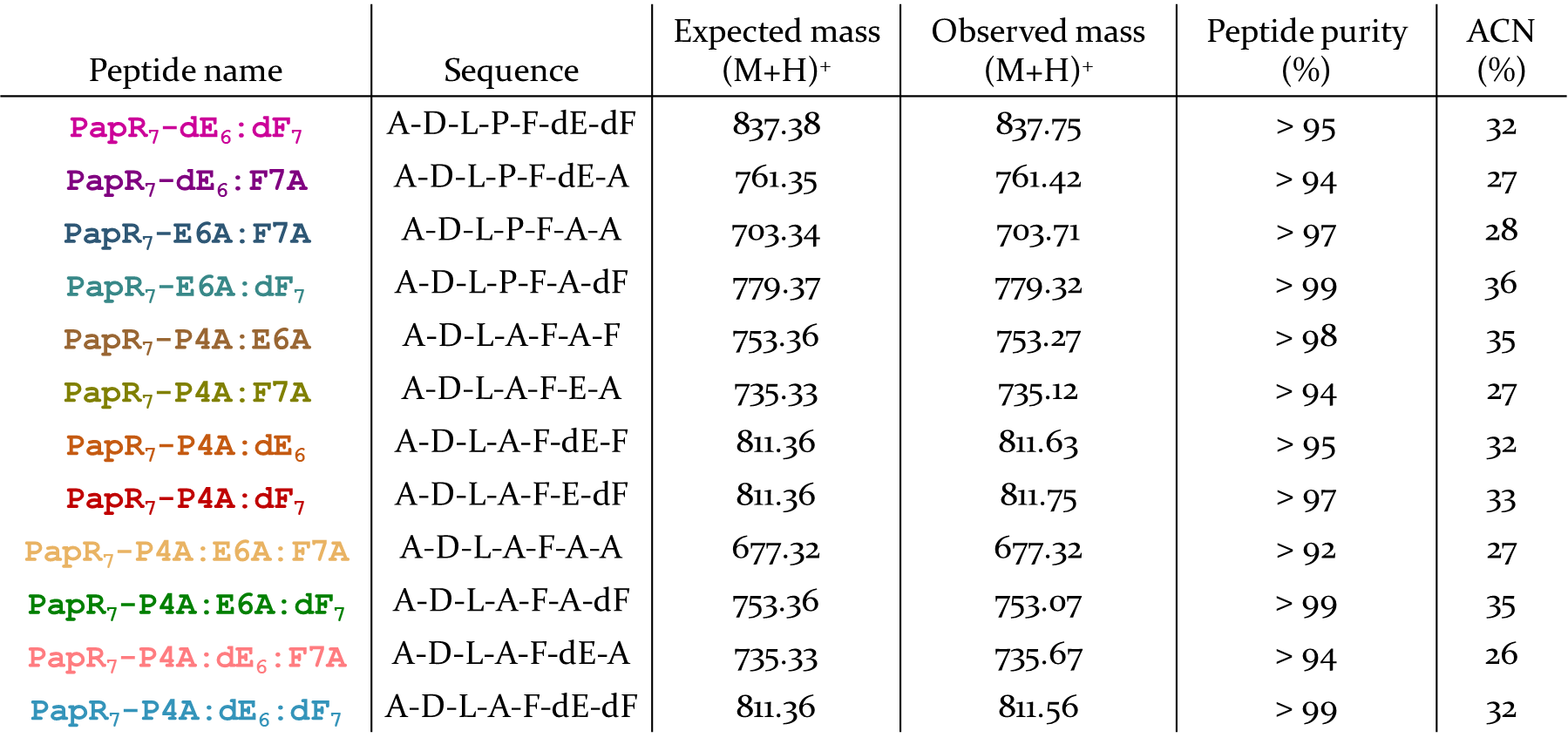
**Table S1:** MS and HPLC data for PapR_7_-derived peptide combinations

**
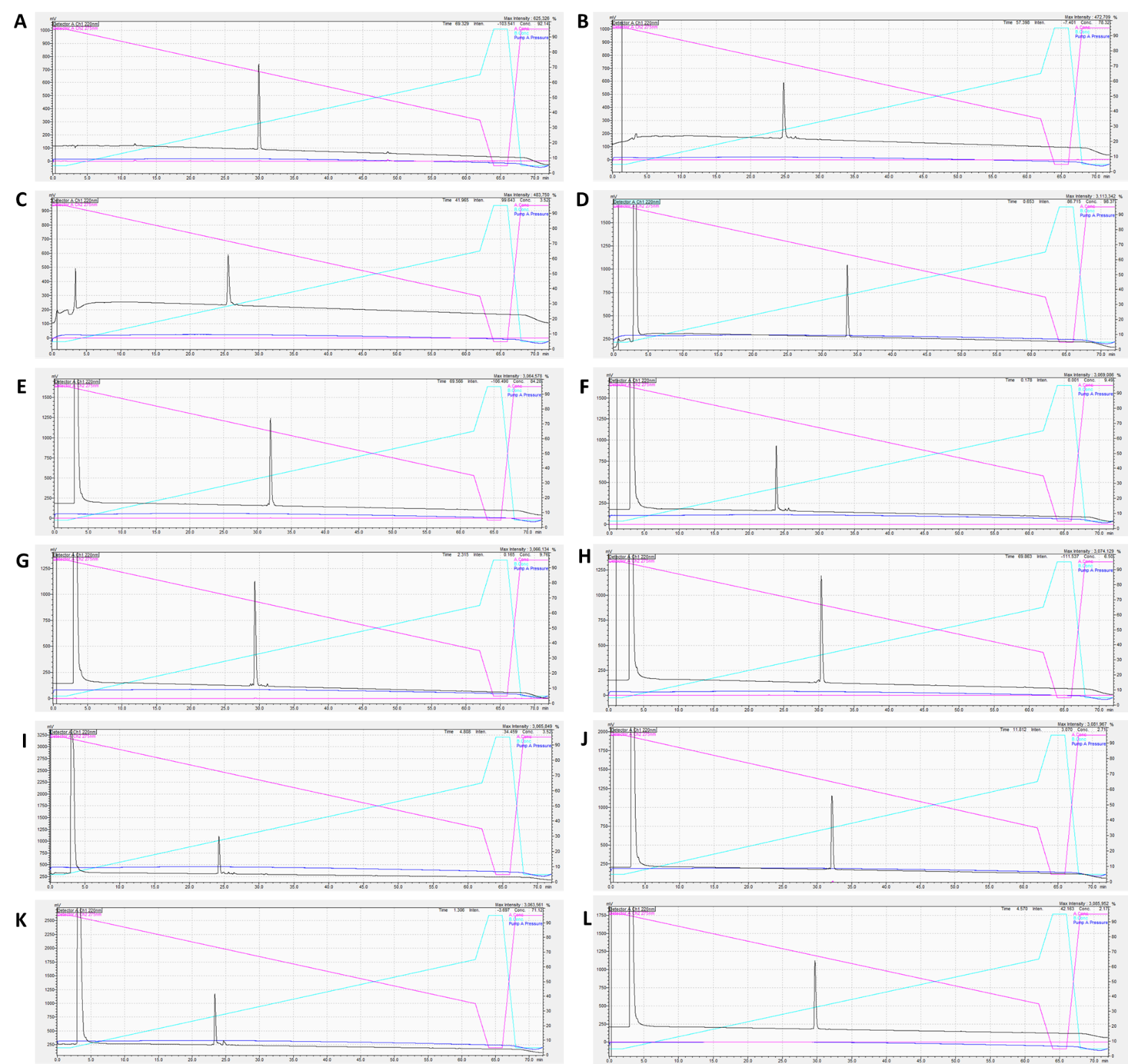
**

**
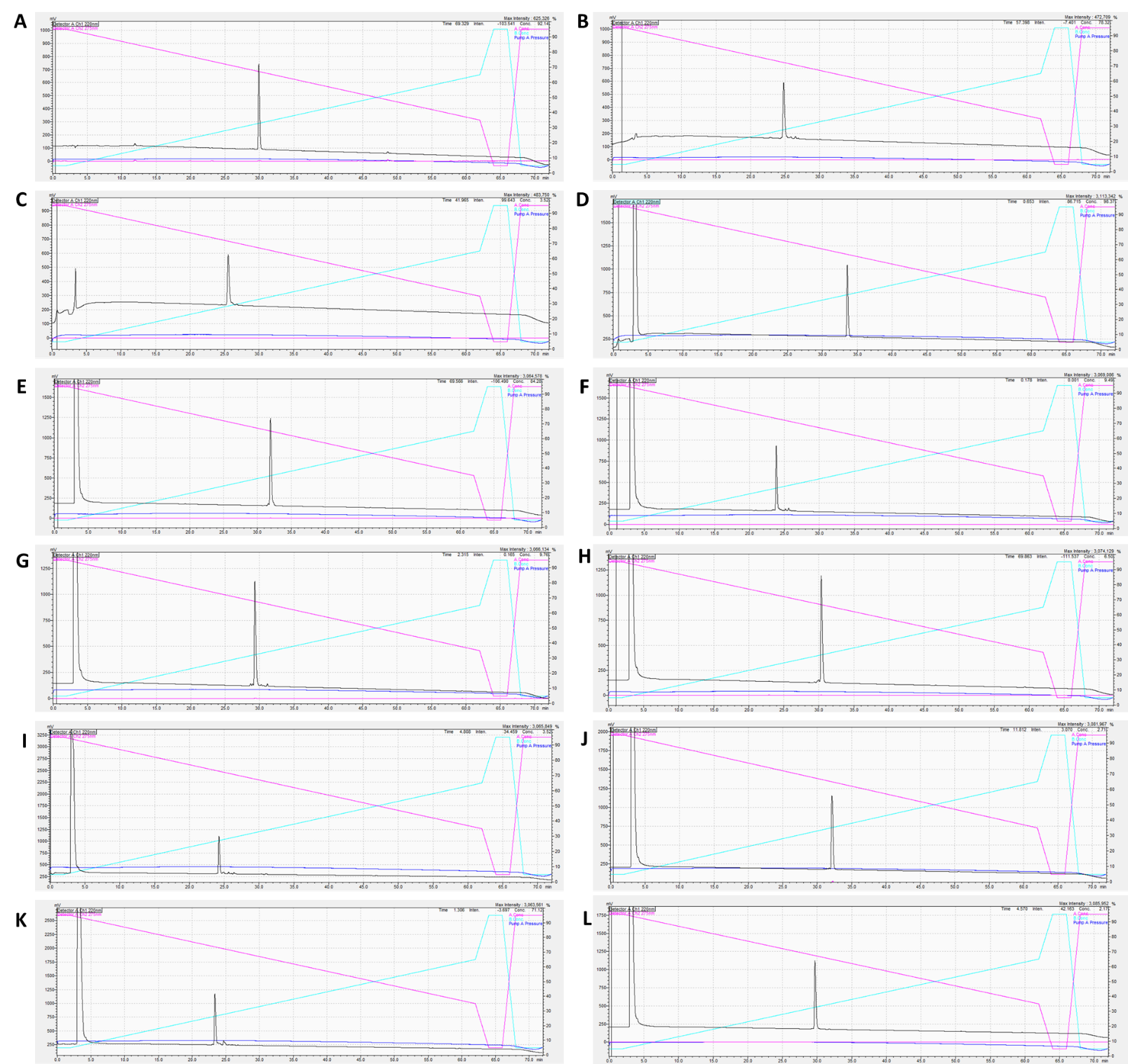
**

**Figure S1: HPLC traces for PapR_7_-derived peptide combinations**. Reverse-phase (RP) high-performance liquid chromatography (HPLC) purification chromatograms of PapR_7_-derived peptide combinations: (A) PapR_7_-dE_6_:dF_7_, (B) PapR_7_-dE_6_:F7A, (C) PapR_7_-E6A:F7A, (D) PapR_7_-E6A:dF_7_, (E) PapR_7_-P4A:E6A, (F) PapR_7_-P4A:F7A, (G) PapR_7_-P4A:dE_6_, (H) PapR_7_-P4A:dF_7_, (I) PapR_7_-P4A:E6A:F7A, (J) PapR_7_-P4A:E6A:dF_7_, (K) PapR_7_-P4A:dE_6_:F7A, (L) PapR_7_-P4A:dE_6_:dF_7_. The gradients of mobile phase solvents are presented; acetonitrile (Cyan) and 18 MΩ water + 0.1% trifluoroacetic acid (Pink).


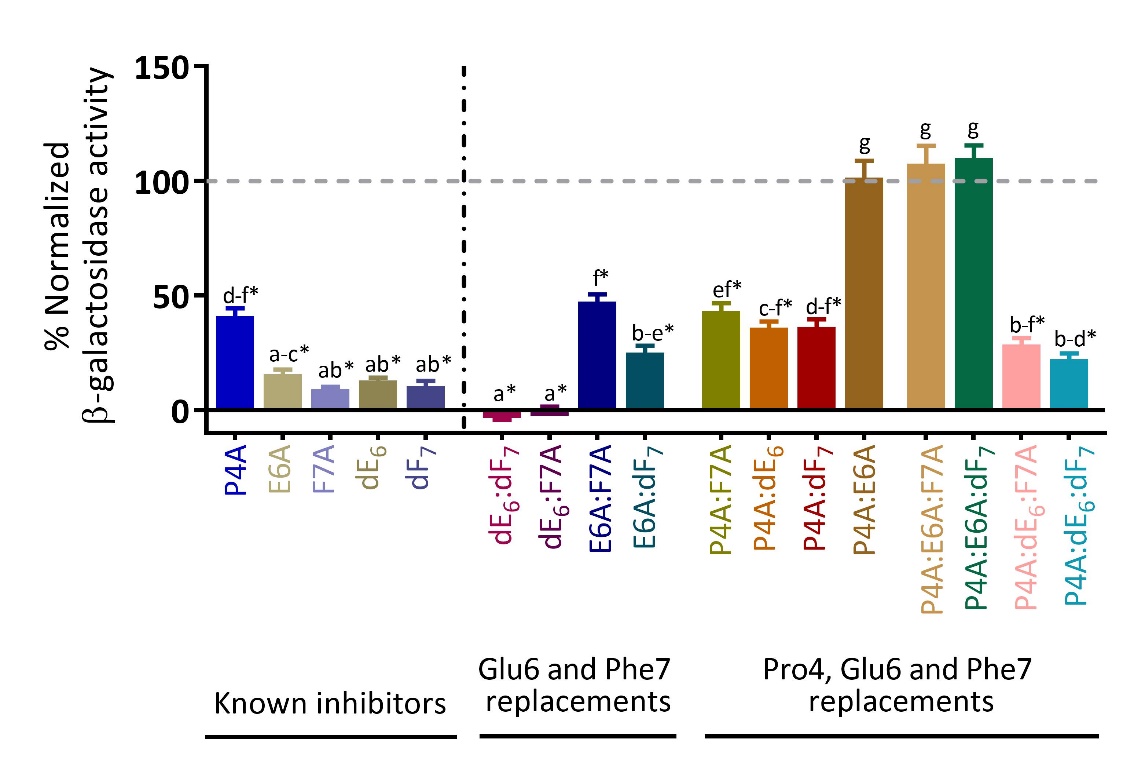


**Figure S2: Blockage of PlcR regulon expression after 24-hour.** β-galactosidase activity of Bt A’Z induced by the addition of 10 µM PapR_7_-derived peptides normalized to untreated bacterial cells at end lag phase of bacterial growth (OD_600_ of 0.1 ± 0.03; mean ± SEM, n = 9). Both treated and untreated bacterial cells were harvested after 24 hr of treatment and β-galactosidase activity was assayed. ^*^p < 0.01 indicates a statistically significant difference between untreated Bt A’Z and addition of PapR_7_-derived peptides. Different letters indicate statistically significant differences between PapR_7_-derived peptide treatments (p < 0.01).
